# Anticancer Effect and Mechanism of Hydroxygenkwanin in Oral Squamous Cell Carcinoma

**DOI:** 10.3389/fonc.2019.00911

**Published:** 2019-09-18

**Authors:** Yi-Chao Huang, Po-Chuan Lee, Jane Jen Wang, Yi-Chiung Hsu

**Affiliations:** ^1^Department of Biomedical Sciences and Engineering, National Central University, Taoyuan, Taiwan; ^2^Department of Medical Laboratory, Taoyuan Armed Forces General Hospital, Taoyuan, Taiwan; ^3^The Department of Nursing, School of Nursing, National Taipei University of Nursing and Health Sciences, Taipei, Taiwan

**Keywords:** oral cancer, RNA sequencing, apoptosis, cell cycle, migration, invasion

## Abstract

The incidence and mortality of oral squamous cell carcinoma (OSCC) are high, and the number of oral cancers had risen in the world. However, chemotherapy drugs have numerous side effects. There is an urgent requirement to develop a novel drug that can be used to treat oral cancer. Hydroxygenkwanin (HGK) is a nature flavonoid extracted from *Daphne genkwa Sieb. et Zucc. (Thymelaeaceae)*. Previous studies had demonstrated that HGK exhibits anticancer effect, but the effect is still unclear in oral cancer. HGK inhibited cell growth dose-dependently in SAS and OCEM1 cells. The functional enrichment analysis showed the significant pathway in cellular movement, cell cycle and cellular growth and proliferation. We further demonstrated the HGK induced the cell cycle arrest by flow cytometry and inhibited colony formation ability and cell movement. The western blot showed that HGK induced cell cycle arrest through p21 activation and caused intrinsic cell apoptosis pathway. HGK inhibited the cell invasion and migration through down-regulation vimentin. HGK might be an effective natural product for oral cancer therapy.

## Introduction

Oral cancer is a lethal disease worldwide with a 5-year survival rate of around 50% and oral squamous cell carcinoma (OSCC) accounts for 90% of all oral cancer types found in the mouth, tongue, and lips (1). The oral cancer is highly related to the exposure of risk factors such as betel nut chewing, smoking, alcohol drinking, and virus infection (2). From 2004 to 2015, the oral cancer incidence keeps increasing in Taiwan. Surgery, radiotherapy, and chemotherapy are the three main treatments intended to stop or eliminate the spread of oral cancer (3). However, chemotherapy compounds would cause normal cells damage. In order to reduce the side effect of treatment in oral cancer, identification of the new potentially anticancer compounds is necessary. Hydroxygenkwanin (HGK) is purification from *Daphne genkwa* plant. *Daphne genkwa* (*yuanhua* in Chinese), is one kind of Daphne plants (*Thymelaeaceae*) distributed in Europe, Taiwan and China. *Daphne genkwa* is a commonly used traditional Chinese medicine (TCM) known to have purgative and diuretic effects (4). The daphnane diterpenoid from the flowers of *Daphne genkwa*, has been demonstrated a potential anti-cancer effect in human non-small cell lung cancer (NSCLC) cells and anti-gioma (5, 6). The *Genkwa flos* flavonoids significantly decreased arthritis through antioxidant and hemorheological modulatory mechanisms in animal study (7). However, the systematic studies of HGK, the compound from *Daphnis Genkwa Flo*, in oral cancer is limited. We systematically study the gene expression profile in OECM1 and SAS cells treated with HGK. Furthermore, we investigate the anti-cancer mechanism of HGK in oral cancer cells.

## Materials and Methods

### Cell Culture

HGK was purchased from Shanghai BS Bio-Tech Co., Ltd (Shanghai, China) and was dissolved in dimethyl sulfoxide (DMSO) (Merck, Darmstadt, Germany) as a stock solution of 100 mM and stored at −20°C before use. SAS is a human tongue squamous cell carcinoma from the Japanese Collection of Research Bioresources (Tokyo, Japan) (2). OECM1, a human gingival squamous carcinoma cells, derived according to previous study (2). Two cell lines were authenticated by short tandem repeat analysis and cultured in Dulbecco's modified Eagle's medium (DMEM) supplemented with 10% fetal bovine serum (FBS), 1.2 g/L sodium bicarbonate, 0.5 mM sodium pyruvate, and 2.5 mM L-glutamine. Chemical compounds, culture medium and FBS were purchased from Life Technologies (Grand Island, NY, USA).

### Phenotypic Examination

Phenotypes including cell proliferation (MTT assay), clonogenic ability, migration, and invasion assay were done as described previously (2, 8).

### RNA Sequencing

The total RNA samples are first treated with DNase I to degrade any possible DNA contamination. Then the mRNA is enriched by using the oligo (dT) magnetic beads. Mixed with the fragmentation buffer, the mRNA is fragmented into short fragments. Then the first strand of cDNA is synthesized by using random hexamer-primer. Buffer, dNTPs, RNase H and DNA polymerase I are added to synthesize the second strand. The double strand cDNA is purified with magnetic beads. End reparation and 3′-end single nucleotide A (adenine) addition is then performed. Finally, sequencing adaptors are ligated to the fragments. The fragments are enriched by PCR amplification. During the QC step, Agilent 2100 Bioanaylzer and ABI Step One Plus Real-Time PCR System are used to qualify and quantify of the sample library. The library products are ready for sequencing via Illumina HiSeq 4000 or other sequencer when necessary.

### Differential Expression Analysis

Primary sequencing data that produced by Illumina Hiseq 4000 (San Diego, CA), called as raw reads, is subjected to quality control (QC) to determine if a resequencing step is needed (Using Trimmomativ v0.33). After QC, raw reads are filtered into clean reads which will be aligned to the reference sequences (mm10 for mouse) by Bowtie2 v2.2.6. QC of alignment is performed to determine if resequencing is needed. The alignment data is utilized to calculate distribution of reads on reference genes and mapping ratio. Once all of the clean reads mapped to reference sequences, we're using “RSEM (RNA-seq by Expectation Maximization)” for calculating read raw count and normalized quantification from each sample. As we got the read quantification data, we could continue various different comparisons. The statistical tool we selected is “EBSeq v1.16.0” which may be used to identify DEGs (differential expressed genes). The RNAseq data were publicly available on NCBI's Sequence Read Archive (SRA) database (Bio-project: PRJNA559691).

### Functional Enrichment

The pathway enrichment were analyzed by the Ingenuity Pathway Analysis (IPA) (QIAGEN company, Redwood City, CA, USA), and Gene set enrichment analysis (GSEA) (9).

### Cell Cycle Analysis

Cell cycle analysis was performed as previously described (10). Briefly, cells were fixed in −20°C absolute ethanol for 4 h and resuspended in PBS having 20 μg/ml ribonuclease A. After incubating at 37°C for 30 min, propidium iodide 100 μg/ml was added to samples. Cell cycle was examined by flow cytometry (BD FACSCalibur TM system, Becton–Dickinson).

### Western Blotting

Western blotting was performed as described previously (2). Antibodies against poly (ADP-ribose) polymerase (PARP) (Asp214), caspase 9, phosphor-H2A.X (ser139), E-cadherin, and Vimentin were purchased from Cell Signaling (Temecula, CA, USA). Anti-p21 and β-actin were purchased from Santa Cruz Biotechnology (Santa Cruz, CA, USA).

### Statistics

Student's *t*-test was completed by the Statistical Package for the Social Sciences version 12.0 (SPSS, Inc). Differences between the variables were considered significant for *p*-values < 0.05.

## Results

### Effects of HGK on the Cell Survival Assay and Colony Formation in SAS and OECM1 Cells

**Figure 1:**
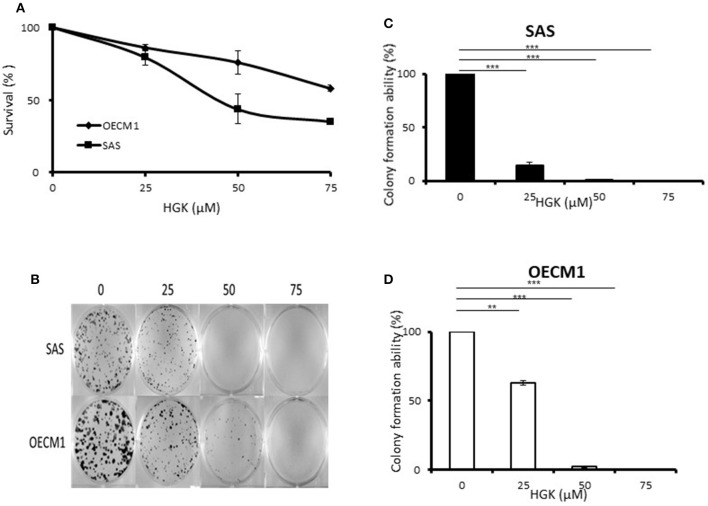
Effects of HGK on the cell survival assay and colony formation of SAS and OECM1 cells. SAS and OECM1 cells were treated with HGK for 24 h in different concentrations (0, 25, 50, 75 μM). **(A)** MTT assay **(B)** The images showing colony formation assay in two cancer cells treated with HGK. **(C)** The quantitative analysis of colony numbers in SAS cells **(D)** in OECM1 cells. **P < 0.01 and ***P < 0.001.

To determine the ability of HGK to inhibit cell growth and colony formation in two cell lines, cells were incubated in the absence or presence of increasing concentrations of HGK for 24 h. The growth and colony formation of SAS and OECM1 cells were reduced after exposure to 25–75 μM HGK for 24 h (Figure 1). The inhibition activity of HGK on cell survival assay and colony formation of SAS cells was more potent than on OECM1 cells.

### Functional Enrichment Analysis of Differential Gene Expression Genes After HGK Treatment

**Figure 2:**
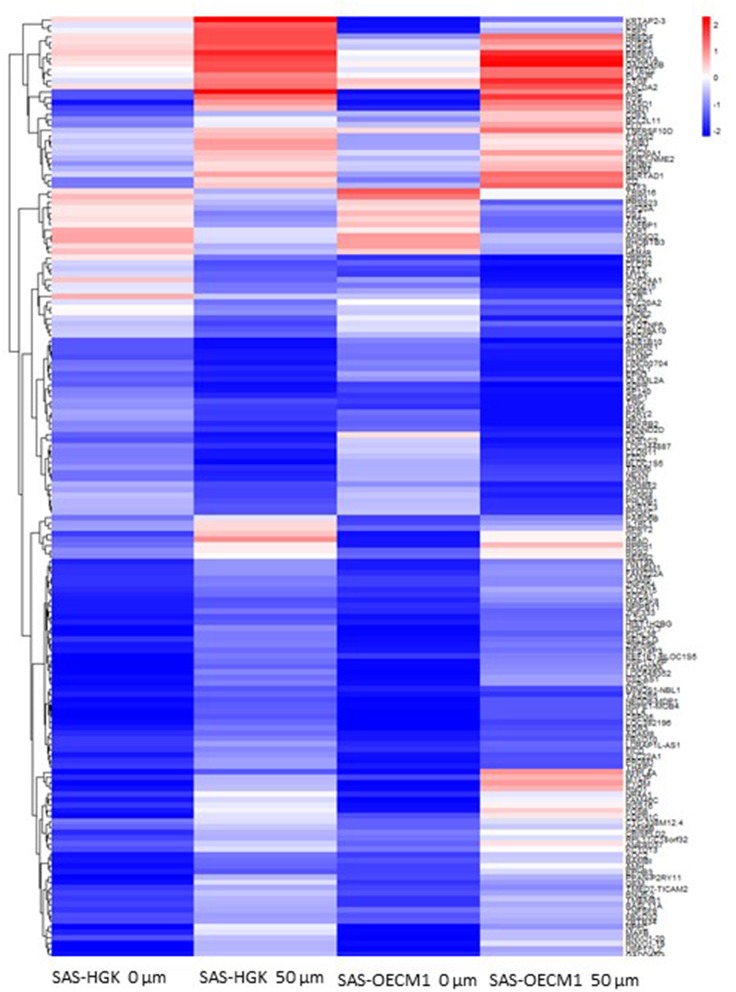
Heatmap showing differential gene expression level with 50 μM HGK treatment for 24 h. Red indicating upregulation, and blue representing downregulation.

**Figure 3:**
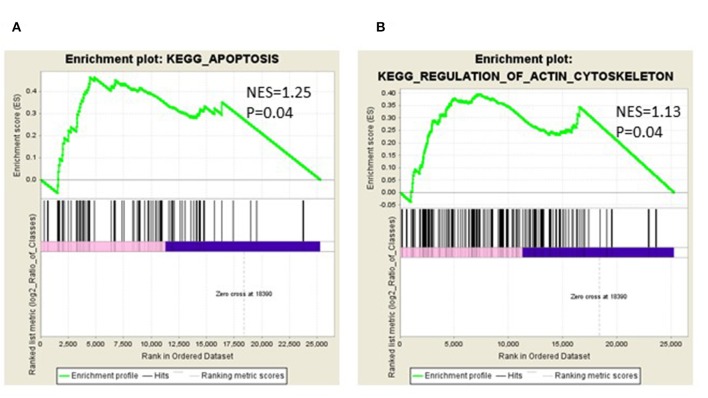
Gene Set Enrichment Analysis (GSEA) enrichment plot involved in **(A)** apoptosis **(B)** in regulation of actin cytoskeleton.

We identified the differential gene expression genes from RNAseq data. A filtering criterion of FPKM value is more than 1 (11), and log2 fold change was greater than two in both SAS and OECM1 datasets. The sign of change must be consistent in both cell lines. A total of 274 genes were significant in the SAS dataset and 969 genes in the OECM1 dataset (Table S1). 170 genes were consistent change in both cell lines (Figure 2). Table 1 showed the most significant molecular and cellular function of differential expression genes after HGK treatment in SAS and OECM1 cells. The final consistency 170 genes were listed in the Table S2. The cellular functions affected by HGK were involved in the cellular movement, cell cycle and cell proliferation (Table 1). GSEA shows a statistically significant difference between biological samples association with apoptosis and regulation of actin cytoskeleton functions (Figure 3). Differential expression and pathway analysis in HGK treatment oral cancer cells indicated the cell cycle, cell survival and cell movement pathways were potential mechanisms.

**Table 1:** Molecular and cellular functions analysis of differential gene expression in HGK-treated oral cancer cells.

**Name**	***p-*value range**	**#Molecules**
Cellular movement	2.32E-04–2.39E-15	68
Cell death and survival	2.34E-04–7.60E-12	74
Cell cycle	2.35E-04–1.09E-11	87
Cellular growth and proliferation	2.35E-04–1.09E-11	78
DNA replication	2.80E-11–2.80E-11	22

### Effects of HGK on the Cell Cycle Progression in SAS and OECM1 Cells

**Figure 4:**
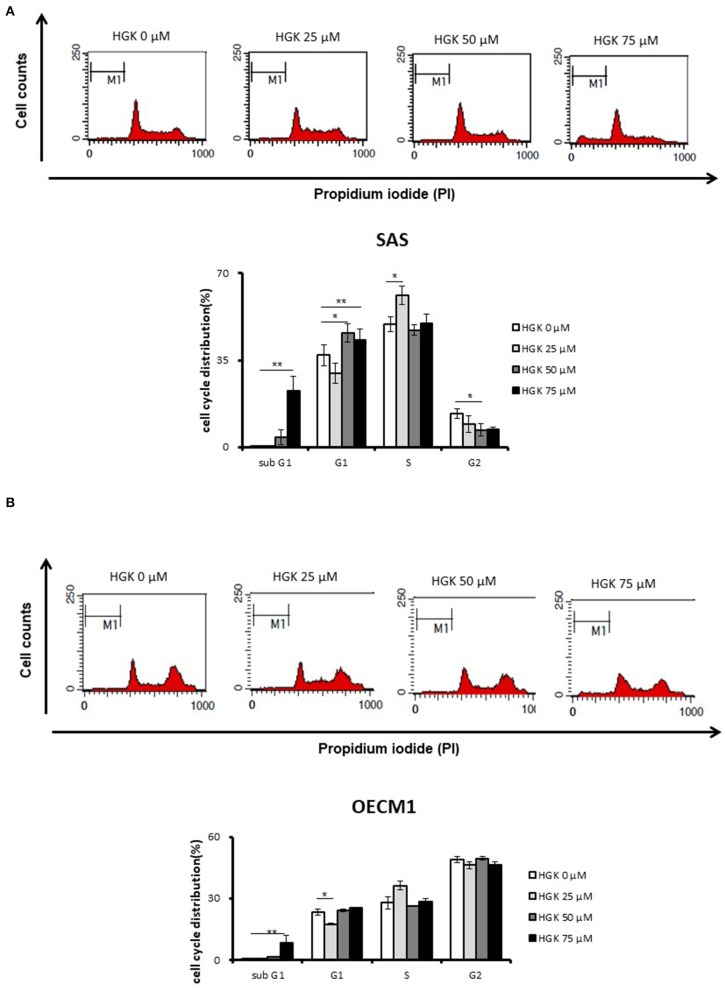
Effects of HGK on the cell cycle progression in SAS and OECM1 cells. SAS cells **(A)** and OECM1 cell **(B)** were treated with 0, 25, 50, 75 μM for 24 h. The results are shown as the mean ± S.E. of three independent experiments. *P < 0.05 and **P < 0.01.

We further examine the HGK biological function according to the results of functional enrichment. For investigation of the effect of HGK on cell cycle progression, cells were stained by propidium iodide (PI) and measured by flow cytometry at 24 h following 25–75 μM HGK drug treatments. In Figure 4, as compare to control (0 μM), HGK dose dependently increased the percentage of sub G_0_/G_1_ phase SAS and OECM1 cells, and reduced the cells in G2/M phases of SAS cells but not OECM1 cells. 50 and 75 μM HGK increased the percentage of G_0_/G_1_ phase SAS cells, but 25 μM HGK reduced both of the cells in G_0_/G_1_ phases and concomitantly increased the percentage of S phase cells (Figure 4).

### Effects of HGK on Cell Motion in SAS and OECM1 Cells

**Figure 5:**
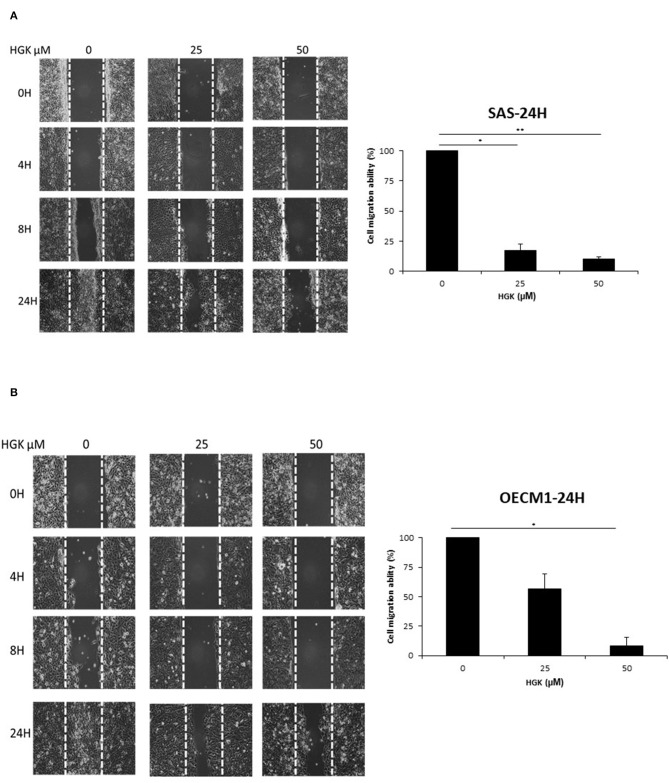
Effects of HGK on cell migration in SAS **(A)** and OECM1 **(B)** cells. SAS and OECM1 cells were treated with 0, 25, 50, 75 μM HGK for 24 h. The results are shown as the mean ± S.E. of three independent experiments. *P < 0.05 and **P < 0.01.

**Figure 6:**
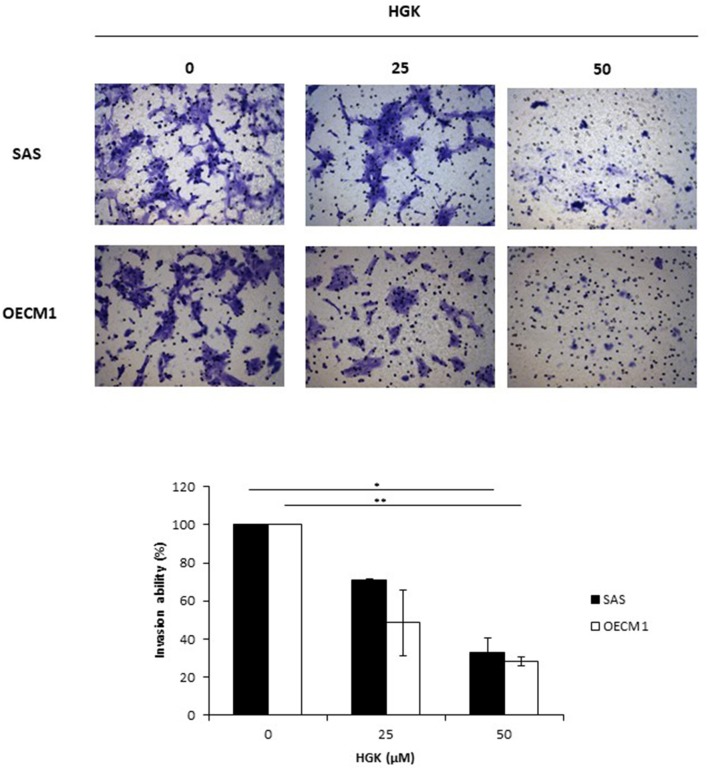
Effects of HGK on cell invasion in SAS and OECM1 cells. SAS and OECM1 cells were treated with 0, 25, 50, 75 μM HGK for 24 h. The results are shown as the mean ± S.E. of three independent experiments. *P < 0.05 and **P < 0.01.

To investigate the HGK effect on SAS and OECM1 cell migration, we used wound healing assay. The cell migration was obviously reduced in SAS and OECM1 cells at 8 and 24 h after 25 and 50 μM HGK treatment (Figure 5). To investigate the HGK effect on SAS and OECM1 cell invasion, we used transwell invasion assay. As shown in Figure 6, the invasion was significantly reduced in 25 and 50 μM HGK treated- SAS and OECM1 cells.

### Effects of HGK on Cell Cycle, Apoptosis, and EMT Regulatory Proteins in SAS and OECM1 Cells

**Figure 7:**
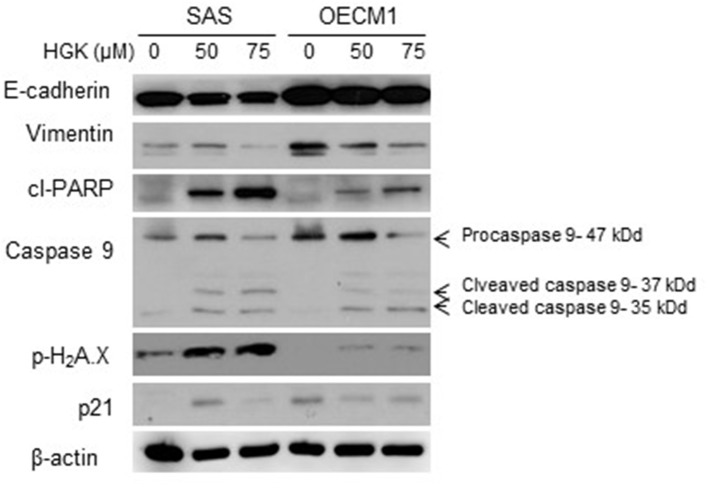
Western blot analysis of cell cycle, apoptosis, and EMT regulatory proteins after treatment with HGK in SAS and OECM1 cells. β-actin was used as the internal marker.

In a previous result, we confirmed that HGK increased the percentage of SAS cells but not OECM1 cells in G0/G1 phase. In order to identify the molecular involved in the HGK effect of the G0/G1 phase, SAS and OECM1 cells were treated with 50 and 75 μM HGK for 24 h, and the cells were harvested for Western blotting. We observed the up-regulation of p21 in SAS cells after HGK treatment, but downregulation of p21 in OECM1 cells (Figure 7). p21 acts at the checkpoint in the cell cycle at the G1 phase. Western blot indicated that cell cycle regulated p21 might play an important role in in HGK-induced cell cycle arrest. HGK dose dependently increased the percentage of sub G_0_/G_1_ phase SAS and OECM1 cells (Figure 4). We further examined the apoptosis effect by using of H2AX Phosphorylation (12) and cleaved PARP cleaved caspase 9 (13). Western blot analyses of pH2AX, cl-PARP and cleaved caspase 9 in both HGK treated-SAS and OECM1 cells. The epithelial–mesenchymal transition (EMT) provides cells with migration and invasion abilities (14). We measured the e-cadherin (epithelial marker), and vimentin (mesenchymal markers) in HGK-treated cells. Vimentin showed significantly decreased between the HGK-treated and control groups, but not significant up-regulation of E-cadherin (Figure 7).

## Discussion

Natural compound and their related moieties played an important role in drug discovery and development (15). HGK was obtained from novel flavonoid extracted from *Daphne genkwa* and induced cancer cell toxicity, cell cycle arrest, apoptosis and EMT signaling pathways in different types of OSCC cell lines (OECM1 and SAS). We demonstrated that HGK significantly dose-dependently inhibited the rate of cell growth in OECM1 and SAS cells. SAS cells were higher sensitivity to HGK than OECM1 cells (Figure 1). The SAS cell line was high malignancy and metastasis derived from a poorly differentiated squamous cell carcinoma. The OECM-1 cell line was less malignancy established from a Taiwanese patient (16). Next-generation sequencing technologies are being widely applied in biomedical research (17). Systems for transcriptome profiling provided the insight into the specific biological pathways. Increases in G0/G1 phase cell cycle arrest with increasing doses of HGK treatments in SAS cells, but not OECM1 (Figure 4). Increase of the sub-G1 phase with various doses of HGK in both SAS and OECM1 cells. Western blot results showed that HGK activated the protein levels of p21in SAS cells, but decrease in OECM1 cells (Figure 7). The results were consistent with cell cycle analysis. The G1-S transition in cell cycle is driven mainly by cyclin-dependent kinase (CDK) 2 that is controlled by abundance of CDK inhibitors: p21 (18). p21 was a key cell cycle regulator that arrests cells in the G1 and G2 phases. Therefore, p21 proteins were involved in HGK-induced the cell cycle arrest in SAS cells. HGK induce the cell death of OSCC cells by apoptosis. The mechanisms of apoptosis are involving two main apoptotic pathways: the extrinsic and the intrinsic pathway (19). HGK induced cell apoptosis through the intrinsic pathway by activation of cleave Caspase 9, and cleave PARP (Figure 7). Histone H2AX phosphorylation was sensitive marker for DNA double-strand breaks (20). We demonstrated that HGK induce DNA damage in both two cell lines. Moreover, H2AX phosphorylation also an essential role in cell cycle arrest regulated by p21 and cell growth (21). HGK induced cell cycle arrest not only through p21 activation but also H2AX phosphorylation. Decrease of E-cadherin and gain of vimentin promoted tumor migration, leading to higher metastatic risk of head and neck squamous cell carcinoma patients (22). E-cadherin was an important therapeutic target for designing anti-tumor drugs (6). Figure 7 showed that SAS1 and OECM1 cells did not loss the E-cadherin expression. Therefore, HGK inhibited the cell invasion and migration through down-regulation vimentin, not increase in E-cadherin.

In this study, we provided the bioinformatics analysis and biological functions experiments of HGK-treated oral cancer cells. Our results showed HGK had anti-cancer activities through multiple mechanisms including inhibition of cellular movement, cell cycle arrest and blocking cell proliferation in oral cancer cells. In conclusion, this study provided evidence that HGK was a potential natural anti-tumor compound for squamous cell carcinoma. However, further pharmacological and investigations *in vivo* are required.

## Data Availability

The datasets generated in the study can be found in the SRA database (https://www.ncbi.nlm.nih.gov/sra) using the following accession numbers: STUDY: PRJNA559691, SAMPLE: OECM_HGK (SAMN12551609), EXPERIMENT: 7 (SRX6701569), RUN: OECM1-4_HHFT2DSXX_L4_R1.fastq.gz (SRR9953208), SAMPLE: OCEM_control (SAMN12551608), EXPERIMENT: 5 (SRX6701570), RUN: OECM1-3_HHFT2DSXX_L4_R1.fastq.gz (SRR9953207), SAMPLE: SAS_HGK (SAMN12551607), EXPERIMENT: 3 (SRX6701571), RUN: SAS-2_HHFT2DSXX_L4_R1.fastq.gz (SRR9953206), SAMPLE: SAS_control (SAMN12551606), EXPERIMENT: 1 (SRX6701572), RUN: SAS-1_HHFT2DSXX_L4_R1.fastq.gz (SRR9953205).

## Author Contributions

Y-CHu and P-CL performed the experiments and analyzed the data. Y-CHu, Y-CHs, and JW drafted the manuscript. Y-CHs contributed to conception and design of the study. All authors have read and approved the final manuscript.

### Conflict of Interest Statement

The authors declare that the research was conducted in the absence of any commercial or financial relationships that could be construed as a potential conflict of interest.

## Supplementary Material

Table S1 The gene expression profiles in SAS and OECM1 treated with HGK.

Table S2 The consistency expression genes.
